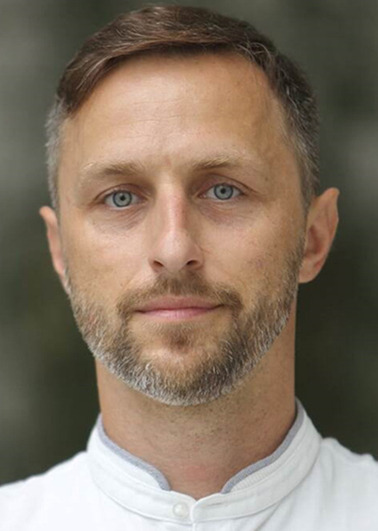# Inter‐relationships between Oxygen Evolution and Iridium Dissolution Mechanisms

**DOI:** 10.1002/anie.202114437

**Published:** 2022-02-09

**Authors:** Anja Lončar, Daniel Escalera‐López, Serhiy Cherevko, Nejc Hodnik

**Affiliations:** ^1^ Laboratory for Electrocatalysis Department of Materials Chemistry National Institute of Chemistry Hajdrihova 19 1000 Ljubljana Slovenia; ^2^ University of Nova Gorica Vipavska 13 5000 Nova Gorica Slovenia; ^3^ Helmholtz-Institute Erlangen-Nürnberg for Renewable Energy Forschungszentrum Jülich Cauerstrasse 1 91058 Erlangen Germany

**Keywords:** dissolution, electrocatalysis, iridium, oxygen evolution reaction, stability

## Abstract

The widespread utilization of proton exchange membrane (PEM) electrolyzers currently remains uncertain, as they rely on the use of highly scarce iridium as the only viable catalyst for the oxygen evolution reaction (OER), which is known to present the major energy losses of the process. Understanding the mechanistic origin of the different activities and stabilities of Ir‐based catalysts is, therefore, crucial for a scale‐up of green hydrogen production. It is known that structure influences the dissolution, which is the main degradation mechanism and shares common intermediates with the OER. In this Minireview, the state‐of‐the‐art understanding of dissolution and its relationship with the structure of different iridium catalysts is gathered and correlated to different mechanisms of the OER. A perspective on future directions of investigation is also given.

## Graphical abstract

The storage of excess renewable energy in the form of hydrogen depends on the efficiency of the oxygen evolution reaction (OER) and thus the rational design of novel iridium‐based catalysts with improved electrocatalytic properties. To achieve this, it is necessary to understand both the OER and dissolution mechanisms as well as their inter‐relationship. This Minireview gathers state‐of‐the‐art information of these phenomena on different Ir catalysts.

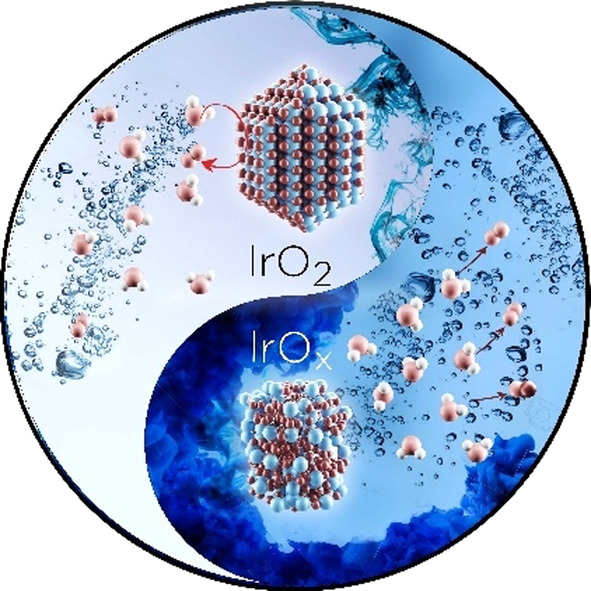

## 1 Introduction

Increasing interest in renewable energy sources, as a result of policies aiming to limit anthropogenic changes to the climate, demands the development of innovative technologies that would mitigate the intermittence of solar and wind energy. Storing excess energy in the form of chemical bonds, specifically as hydrogen, is currently considered one of the best options, which is confirmed by the increasing number of electrolyzer installations worldwide.^[1]^ However, efficient scale‐up of this technology is currently still hindered by the low efficiency, which arises predominantly from the sluggish kinetics of the anodic oxygen evolution reaction (OER) and the high cost of iridium—the only metal that is able to resist the harsh, acidic conditions in the proton‐exchange membrane (PEM) electrolyzers while showing a relatively high activity.[^2^, ^3^] A fundamental understanding of the mechanisms driving the reaction and degradation is of great importance for improving the performance of catalysts. Systematic studies of the OER mechanism on different noble metals can be traced back to the 1950s, when the first kinetic studies were published.[^4^, ^5^, ^6^, ^7^, ^8^] They revealed various possible reaction pathways, which lead to different performances of the Ir catalysts. The focus of investigations on the properties of the Ir catalysts has only recently started to slowly shift from activity towards stability. In this regard, the dissolution of active material is acknowledged as the predominant degradation mechanism. The understanding of the dissolution kinetics has advanced with the development of experimental techniques, such as inductively coupled plasma mass spectrometry (ICP‐MS), which combine electrochemical measurements with the on‐line detection of dissolved species.[^9^, ^10^] The dissolution kinetics are affected by both the operational parameters and physicochemical properties of the catalyst. Specifically, in the case of iridium, the less active rutile (IrO_2_) form is known to be stable under the OER conditions, whereas its amorphous analogues exhibit higher activity for oxygen evolution, but also lower stability. From the dissolution studies, it is apparent that the OER and dissolution mechanisms are intertwined through a common reaction intermediate.[^11^, ^12^] Additionally, the participation in the OER of oxygen from the oxide lattice can lead to destabilization of the Ir‐oxide structure and result in dissolution.

As a consequence of the interlinking of the two reactions, we start this Minireview with a brief discussion on the mechanism of the OER, which has been studied in further depth in other recent reviews.[^13^, ^14^, ^15^] This sets our starting point for a more detailed analysis of the state‐of‐the‐art understanding of the dissolution mechanisms of different Ir‐based catalysts—the core focus of this Minireview—which is generally overlooked in other overarching activity‐ and stability‐oriented publications.[^16^, ^17^] The summary of the most recent literature is concluded with a perspective on the future challenges and strategies to overcome them.

## 2 Mechanism of the Oxygen Evolution Reaction on Ir‐Based Catalysts

**Figure 1:**
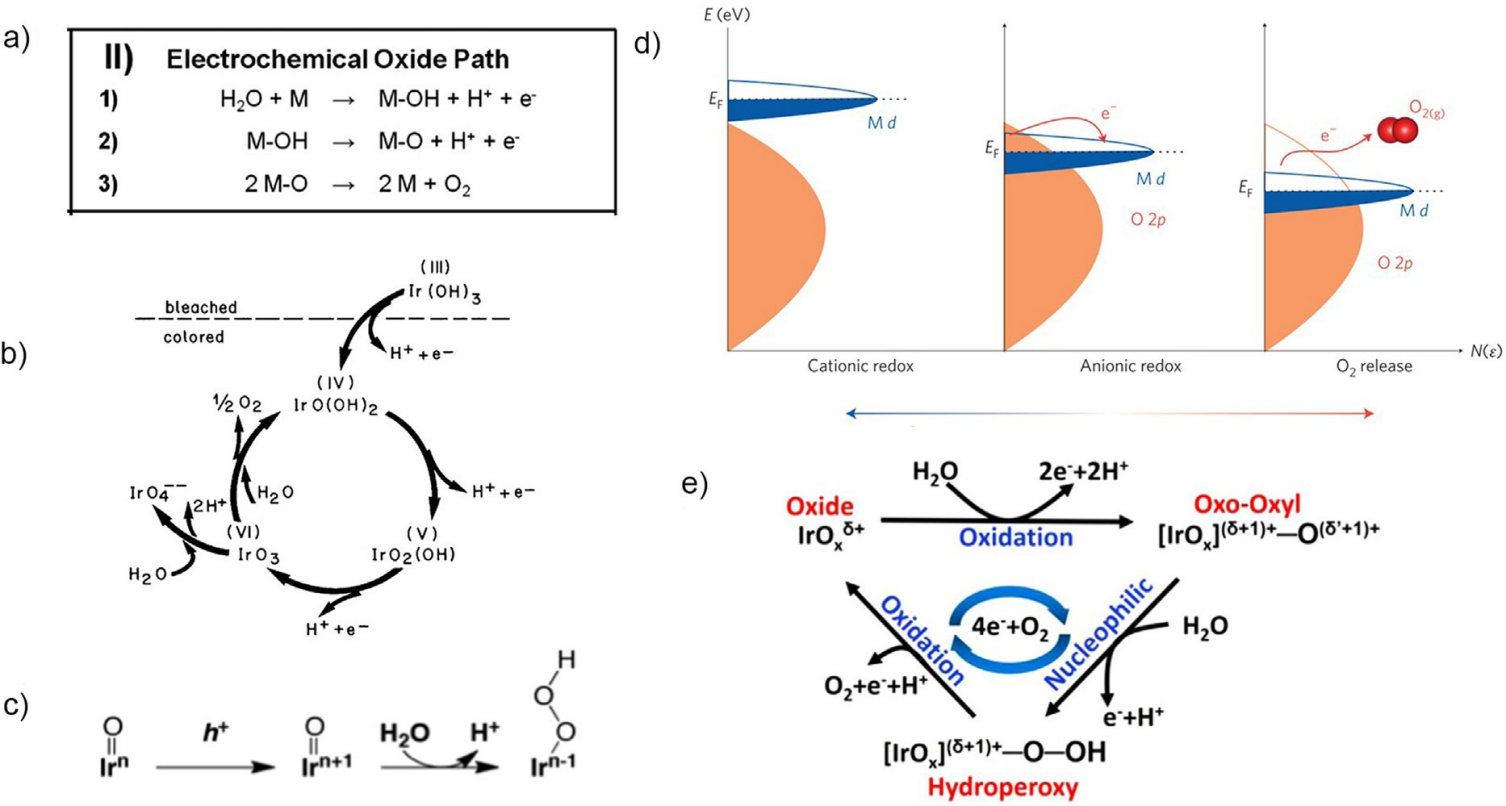
a) Electrochemical oxide pathway.^[13]^ Copyright 2016, John Wiley and Sons. b) Cationic redox mechanism proposed by Kötz et al.^[21]^ Copyright 1984, IOP Publishing. c) Scheme of the OER, including the formation of an OOH intermediate, as detected by Sivasankar et al.^[24]^ Copyright 2011, American Chemical Society. d) Schematic representation of O 2p bands penetrating into Ir d orbitals and triggering an anionic redox process.^[27]^ Copyright 2016, Springer Nature. e) OER scheme showing the formation of oxyl species, as a result of hybridization of Ir and O orbitals, which are prone to nucleophilic attack by water and the formation of an O−O bond.^[33]^ Copyright 2019, Elsevier.

The OER is a complex electrochemical reaction involving four electron and proton transfers and at least two reaction intermediates.^[18]^ The first experimental studies of the OER on different noble metals, which relied only on electrochemical methods such as Tafel analysis, deduced that the OER on iridium occurs by an electrochemical oxide pathway,[^19^, ^20^] which was suggested by Bockris (Figure 1 a).^[4]^ Since then, various studies have aimed to broaden the understanding of the mechanistic pathways, each using a different approach.

Kötz et al. published one of the first ex situ X‐ray photoemission spectroscopy (XPS) studies of IrO_2_ films and, based on the constant ratio between oxygen and iridium at all the studied potentials, proposed a cationic catalytic cycle involving an electrochemical oxide pathway, where iridium is oxidized to an Ir^VI^ intermediate, which is reduced in the following reaction step back to Ir^IV^ with a simultaneous release of an oxygen molecule (Figure 1 b).^[21]^ More recent in situ XPS^[22]^ and X‐ray absorption spectroscopy (XAS)^[23]^ studies on iridium oxide nanoparticles and hydrated iridium oxide films, respectively, have, in contrast, suggested a Ir^V^–Ir^III^ transition, with the presence of both oxidation states under the OER conditions.^[23]^ This mechanism was further supported by Sivasankar et al., who for the first time detected an Ir‐OOH intermediate by probing iridium oxide nanoclusters using infrared (IR) spectroscopy (Figure 1 c).^[24]^ Cationic redox processes, on which these studies were based, have traditionally been thought to be the main source of charge storage.

This view was challenged by the discovery of anion‐driven capacity storage in Li‐ion battery technology.[^25^, ^26^] The idea of an anionic redox mechanism was soon applied to the field of electrocatalysis. It was demonstrated that shifting the p‐band of the oxygen atom closer to the Fermi level in metal oxides with a highly covalent network can trigger the redox activity of the lattice oxygen atoms (Figure 1 d).^[27]^ The anionic redox mechanism was experimentally uncovered in different studies.[^28^, ^29^, ^30^, ^31^] Saveleva et al. aimed to reveal a universal mechanism by using near ambient pressure XPS (NAP‐XPS) and XAS, combined with ab initio calculations, to study two different Ir‐based catalysts: thermally oxidized IrO_2_ and electrochemical amorphous iridium oxide nanoparticles.^[30]^ The authors detected the formation of oxyl O^−^ species on both rutile and the structurally more flexible IrO_*x*_ nanoparticles, and concluded that the involvement of anionic lattice oxygen atoms in the OER is universal, regardless of the structure of the catalyst.

The investigation of oxidation‐state changes of the catalyst is important, as they are directly related to the charge‐storage mechanism, which was recently shown to be the driving force of the reaction.^[32]^ Nevertheless, the latest studies, which employ combinations of in situ spectroscopic techniques and theoretical calculations, are now showing that focusing entirely on either a cationic or anionic mechanism probably does not describe the full picture. It has been shown that, as a result of a strong hybridization of the iridium and oxygen orbitals, the positive charge is shared between cations and anions.^[33]^ Specifically, the formation of the reactive oxyl species depends on the oxidation state of the iridium.^[34]^ As the catalyst is exposed to a high anodic potential, the accumulation of positive charge in electron‐deficient oxygen species results in a decrease of the activation energy for the nucleophilic attack of water molecules and the formation of an O−O bond, which is currently understood to be the rate‐determining step of the OER (Figure 1 e).[^18^, ^32^, ^34^, ^35^]

## 3 Mechanistic Understanding of Ir Dissolution in the OER

**Figure 2:**
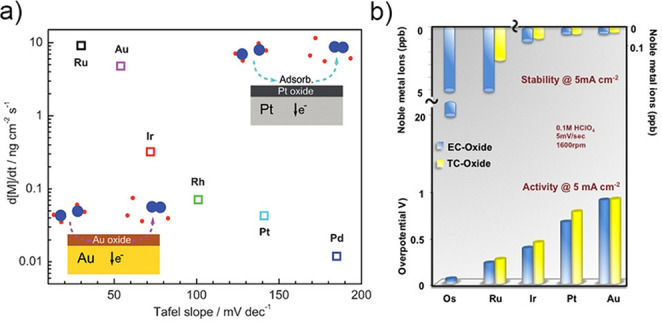
a) Correlation between the stability of the catalyst with a Tafel slope and the operating OER mechanism.^[2]^ Copyright 2014, John Wiley and Sons. b) Activity‐stability trend for different metal oxides, showing an inverse relationship between the reactivity in the OER and the durability of the catalyst.^[37]^ Copyright 2014, American Chemical Society.

One of the first dissolution studies comparing different noble metals, which used the newly developed scanning flow cell (SFC) coupled with inductively coupled plasma mass spectrometry (ICP‐MS),[^9^, ^36^] showed that dissolution of all the studied metals increases as the rate of the OER accelerates, which already suggests a direct interconnection between the activity and stability of OER catalysts.^[2]^ The extent of dissolution was, however, very different depending on the nature of the investigated metal. Iridium was found to have an intermediate response, both in terms of activity and stability. The authors showed that the stability can be estimated from the Tafel slopes, which indicate different operating OER mechanisms. On metals with a high Tafel slope, such as platinum, the OER is expected to proceed through an adsorbate route, which does not involve the participation of the oxide lattice in the reaction and does not disturb the surface of the catalysts, while on metals with a smaller Tafel slope, the OER involves the participation of a thick oxide layer, which results in its destabilization and higher dissolution of the catalyst (Figure 2 a). Interestingly, the authors did not find a correlation between the onset of the OER and dissolution. This implies that the activity and stability of metals are not necessarily in a reverse relationship and that a highly active and durable catalyst could potentially exist. Such a finding is, however, not in agreement with the conclusions from a similar study published the same year that compared the activity and stability of five different metal oxides (Figure 2 b).^[37]^

In this case, the authors observed trends in the OER overpotential and the dissolution of metal cations and suggested that a strong link exists between the two parameters. Furthermore, by comparing highly defective polycrystalline Ru and Ir electrodes to single‐crystalline model electrodes with a well‐defined surface, they deduced that the nature of the oxide and surface defects control the activity and stability of the catalyst. Based on the results, they concluded that since activity and stability are inversely related, the ideal OER catalyst should thus display a balance between them by dissolving “neither too fast nor too slow”.

**Figure 3:**
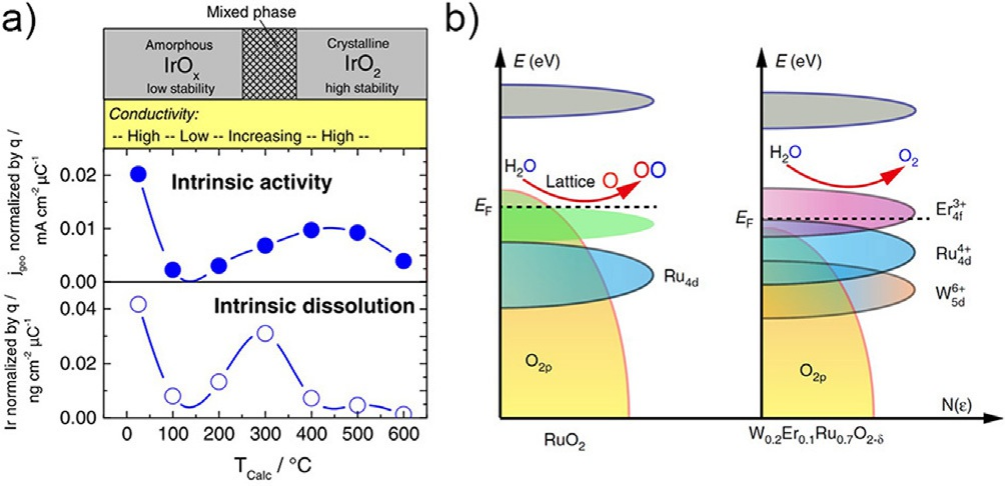
a) Effect on the activity and stability of thermally treating hydrous iridium oxide films.^[39]^ b) Stabilization of the RuO_2_ lattice by increasing the formation energy of oxygen vacancies.^[40]^

The dissolution of different iridium oxides was further tested to determine which parameters result in the improved stability of different oxides. It was shown that thermal treatment of either chemically^[38]^ or electrochemically^[39]^ prepared iridium oxides resulted in improved stability and decreased activity. This trend was explained by changed stoichiometries and increased crystallinity after annealing. The lower dissolution resistance of oxides heat‐treated between 100 and 300 °C showed that, besides the crystal structure, the hydration and conductivity of the oxide play a significant role in the activity–stability properties (Figure 3 a). The effect of different crystal structures was further shown in a study investigating the activity and stability of iridium and ruthenium as well as their oxides.[^40^, ^41^, ^42^] As discussed above, the participation of oxide in the OER promotes the dissolution of the catalyst. In a recent publication, Hao et al. showed that the active involvement of lattice oxygen can be manipulated by tuning the electronic structure of the catalyst. The involvement of lattice oxygen in O−O bond formation can be suppressed by changing the formation energy of oxygen vacancies (*V*_O_; Figure 3 b).^[40]^ Practically, this hypothesis was demonstrated by doping the RuO_2_ lattice with W and Er. This approach was shown to be exceptionally effective, as the downshift in the oxygen 2p band resulted in the representative catalyst displaying long‐term dissolution stability over at least 500 h. After analyzing the aforementioned study, Exner pointed out that calculating *V*_O_ could potentially be used in future theoretical studies as a stability metric.^[43]^

The need for universal stability descriptors is particularly relevant for the experimental evaluation of novel materials. It is well‐documented that parameters such as the accumulation of oxygen bubbles^[44]^ or passivation of the backing electrode^[45]^ can lead to erroneous interpretations of traditional stability measurements with techniques such as rotating disc electrodes (RDEs). Additionally, the choice of experimental parameters, that is, dynamic or stationary measurement procedures,^[46]^ loading effect, and the unknown active surface area of the catalyst further complicate the dissolution and thus stability evaluation of the investigated material. To overcome such limitations, novel metrics such as the S‐number^[47]^ and equivalent activity‐stability factor (ASF)^[42]^ are emerging. Both are defined as the ratio between the number of evolved oxygen molecules and dissolved iridium atoms. These metrics explicitly show the correlation between the activity and stability of different catalysts, which can be used to compare the different stabilities of newly designed materials.[^48^, ^49^, ^50^, ^51^, ^52^, ^53^] However, it is important to emphasize that unifying the experimental parameters is crucial, as different protocols, that is, cycling or potential, affect the dissolution by triggering transient or steady‐state dissolution, and thus affect the S‐numbers.[^2^, ^54^]

To further elucidate the dissolution of iridium over a broader potential range, a series of systematic examinations of the dissolution of both a bare metallic iridium disk and electrochemically grown iridium oxide were carried out by Cherevko et al.[^11^, ^55^] It was shown that the dissolution of hydrous iridium oxide depends both on the potential of the electrode and the thickness of the oxide layer; an increase in both parameters leads to enhanced dissolution. By combining the experimental data with the OER mechanisms already proposed in the literature,[^21^, ^23^] the authors suggested that Ir might dissolve via either Ir^III^ or Ir^VI^ intermediates, depending on the structure of the catalyst.

**Figure 4:**
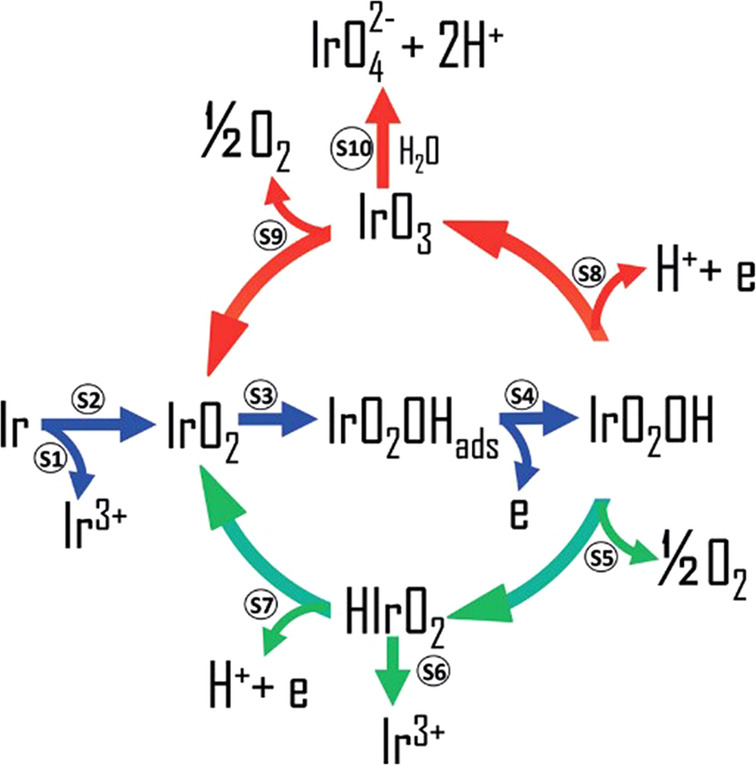
Universal mechanism correlating both the OER and dissolution pathways proposed by Kasian et al.^[12]^ Copyright 2018, John Wiley and Sons.

To confirm this hypothesis, Kasian et al. combined dissolution measurements obtained using SFC‐ICP‐MS with the detection of volatile intermediates and products of the OER by online electrochemical mass spectrometry (OLEMS). By investigating three different iridium anodes, namely metallic iridium as well as reactively sputtered and thermal iridium oxide, the authors detected Ir^VI^ intermediates for the first time.^[12]^ They simultaneously measured the dissolved iridium and formation of O_2_ and IrO_3_ at 5, 10, 15, and 20 mA cm^−2^. On thermal oxide, which displays a lower reactivity towards the OER, the formation of the volatile IrO_3_ intermediate was already detected at the lowest current density, whereas on the other two more‐active electrodes it was possible to detect it only at the highest current densities after the potential on the anode exceeded 1.6 V. The state‐of‐the‐art understanding of the OER and dissolution were combined into a potential‐dependent universal scheme, centered on a cationic redox mechanism (Figure 4). Regardless of the material, the first step of the OER, marked with blue arrows, is the elimination of water and adsorption of an OH radical on the surface of the catalyst, which is accompanied by the oxidation of the iridium center and leads to the formation of the Ir^V^O_2_(OH) intermediate. The next steps depend on the electrode potential, which is determined by the nature of the electrode. If the OER is catalyzed by thermal oxide, the required potential is high enough for further oxidation of iridium to Ir^VI^O_3_. This intermediate can then either decompose to O_2_ and IrO_2_ to close the catalytic cycle or react with water and dissolve as IrO_4_^2−^. Considering the relatively low dissolution of the thermal oxide, it was suggested that hydrolysis is kinetically suppressed, which could explain the superior stability of crystalline iridium oxide. In the case of more‐active materials, the applied potential is not high enough to further oxidize the iridium. Instead, the OER cycle is closed by decomposition of the Ir^V^O_2_(OH) intermediate with the evolution of an O_2_ molecule and formation of the HIr^III^O_2_ species, which can either dissolve as Ir^3+^ or be further oxidized to IrO_2_. A previous study by Cherevko et al.^[11]^ already suggested the formation of this Ir^III^ intermediate to be the origin of the lower stability of metallic and hydrous iridium oxide catalysts. When the current densities are high enough to exceed the potential required for the oxidation of iridium further to Ir^VI^, the pathway marked with red arrows also becomes relevant for the more‐active catalysts. Here, dissolution through the formation of IrO_4_^2−^ might, however, not be equally hindered kinetically, since Geiger et al. showed that, at potentials above 1.8 V, metallic iridium was already completely dissolved after 10 minutes.^[47]^

Assuming that the proposed mechanism is operative, two different dissolution pathways, accompanying the OER, are possible for different catalysts. The first dissolution route involves the unstable Ir^III^ intermediate, which is formed on more active catalysts, such as hydrous iridium oxide and metallic iridium. The involvement of this intermediate was experimentally proven before,[^22^, ^23^] and is usually assumed to be the reason for the poor stability of these materials. Based on the observed high level of dissolution, it was concluded that the dissolution of Ir^III^ is kinetically faster than further oxidation to IrO_2_. The second dissolution pathway involves the hydrolysis of IrO_3_. The third route, which should be mentioned as it also leads to degradation of catalyst but is, however, not directly related to the OER, is the inevitable dissolution of metallic iridium that accompanies the formation of protective passive oxide on the surface of a metal when it is exposed to high anodic potentials.^[56]^ The extent of dissolution depends on the nature of the metal, specifically on the cohesive energy and adsorption energy of oxygen,^[57]^ as well as the surface structure.^[58]^ The relevance of the proposed general mechanism was evaluated by ab initio molecular dynamics simulations.^[59]^ The authors confirmed the thermodynamic stability of the Ir^V^ intermediate over a relatively broad potential window, with the Ir^V^ intermediate being further transformed into IrO_3_ at high anodic potentials and HIrO_2_ at lower potentials. When further simulating the detachment of the Ir^III^ intermediate from the surface, the authors found that iridium can either deposit back on the surface of the oxide or dissolve as Ir(OH)_3_, with the kinetics of redeposition being faster than the dissolution. Amorphization of the surface was experimentally observed and is thus in line with the calculations.[^60^, ^61^] When considering the second dissolution route through IrO_3_ formation, it was shown that the reactivity of this intermediate towards the OER was higher than that of the IrO_2_(110) surface.

Interestingly, no mechanistic study published to date has correlated dissolution with the anionic redox process. Velasco‐Velez et al. recently showed that the anionic mechanism, that is, active participation of O^−^ in the OER, depends on the presence of electron‐deficient Ir^V^ sites in IrO_*x*_.^[34]^ In the future, such studies should also be done on rutile nanoparticles, as the formation of Ir^VI^, as detected by Kasian et al.,^[12]^ still needs to be explained in the context of an anionic redox process. Saveleva et al. detected O^−^ in rutile and concluded that this electrophilic species might be an intermediate of the OER, regardless of the structure of the catalyst.^[30]^ Looking from both perspectives, it might be suggested that, due to strong hybridization between the oxygen and iridium orbitals, the anionic and cationic mechanisms cannot be discussed separately. Nevertheless, if it is assumed that the O^−^ intermediate is stable, the scheme proposed by Kasian et al. is still feasible and will only need to be complemented with O^−^ containing intermediates of an OER catalyzed by iridium oxides.

The presence of unstable intermediates promotes the dissolution of catalysts during the OER. Based on the possible presented reaction pathways, it can be concluded that the OER and dissolution are two parallel reactions with a common intermediate. With this in mind, it may be possible to suppress one without impacting the other. However, the dissolution of the Ir^III^ or Ir^VI^ intermediates is not fully understood and it is not yet known whether the reaction is chemical or electrochemical. Nevertheless, the formation and lifetime of intermediates depend on the potential. As the OER progresses, the concentration of protons in the pores of the oxide can significantly increase, which could lead to enhanced dissolution of the less‐stable intermediates. Finding the conditions where dissolution would be suppressed is, therefore, crucial to forthcoming stability‐related studies. This could be achieved, for example, with a change in the pH value of the electrolyte. Additionally, the stabilization of the IrO_3_ intermediate has already been demonstrated experimentally, specifically through a proton intercalation mechanism.^[62]^

## 4 Lattice Oxygen Evolution Reaction and Its Implications on the Stability of Ir Oxides

**Figure 5:**
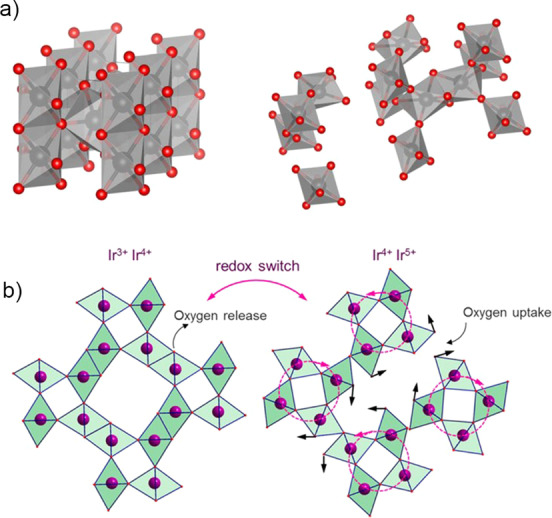
a) Ordered rutile structure with edge‐sharing octahedra and hydrated amorphous structure with activated corner‐sharing oxygen atoms.^[47]^ Copyright 2018, Springer Nature. b) “Paddle wheel” reaction scheme, proposed by Willinger et al.^[69]^ Copyright 2017, American Chemical Society.

The participation of lattice oxygen was mentioned repeatedly in the previous sections. It has been shown that the less‐stable catalysts, such as Ru and Au, are covered by a thick layer of surface oxide, which actively participates in the reaction.[^63^, ^64^, ^65^] However, it should be noted that this process was found to be structure‐dependent.^[66]^ The involvement of the lattice is known to trigger enhanced dissolution.^[2]^ The first study, which quantified the extent of the involvement of the oxide layer in the OER on Ir, was published by Fierro et al.^[67]^ The authors combined differential electrochemical mass spectrometry (DEMS) and isotope labeling to detect oxygen that evolved from the oxide layer in Ti/IrO_2_. Through the detection of species with *m*/*z* 32 and 34, it was confirmed that oxygen is indeed partially evolved from the lattice. However, the study did not correlate this phenomenon with the possible destabilization of the structure through the formation of vacancies after the release of oxygen atoms. Recently, two studies extended this experimental approach with additional simultaneous dissolution measurements.[^47^, ^68^] Geiger et al. examined the stability of different Ir‐based catalysts, namely highly active perovskites, amorphous IrO_*x*_, metallic iridium, and rutile IrO_2_. They found that the rate of dissolution depends on the structure of the catalyst. Non‐noble elements present in the perovskites dissolved immediately after immersion in the acidic electrolyte, leaving behind amorphous, highly hydrated iridium oxide resulting from the collapse of the originally present iridium octahedral framework. The ordered structure with predominately edge‐sharing oxygen atoms transformed into an amorphous structure with an increased number of corner‐sharing oxygen atoms, which resulted in the enhanced dissolution of iridium. Additional isotope‐labeling experiments on rutile and hydrous iridium oxide thin films provided information on the involvement of the activated, corner‐sharing oxygen atoms in the mechanism of the OER (Figure 5 a). The authors concluded that the involvement of lattice oxygen in the OER depends on the structure and that the overall stability of different oxides is determined firstly by the stability of intermediates, which could be higher for the rutile structure compared to the amorphous oxides, and secondly by the ratio between the edge‐ and corner‐sharing iridium octahedra, which was also in line with the observations of Willinger et al.^[69]^ Here, STEM and EELS analysis gave insight into the structural origin of the high activity of amorphous oxides. The authors observed the presence of interconnected hollandite‐like motifs, in which oxygen is evolved through a so‐called “paddle‐wheel” mechanism (Figure 5 b). Additionally, they assumed that the presence of K^+^ ions in the active phase could stabilize the open hollandite structure.

### 4.1 Lattice Oxygen Evolution Reaction and Stability of Hydrous IrO_x_

**Figure 6:**
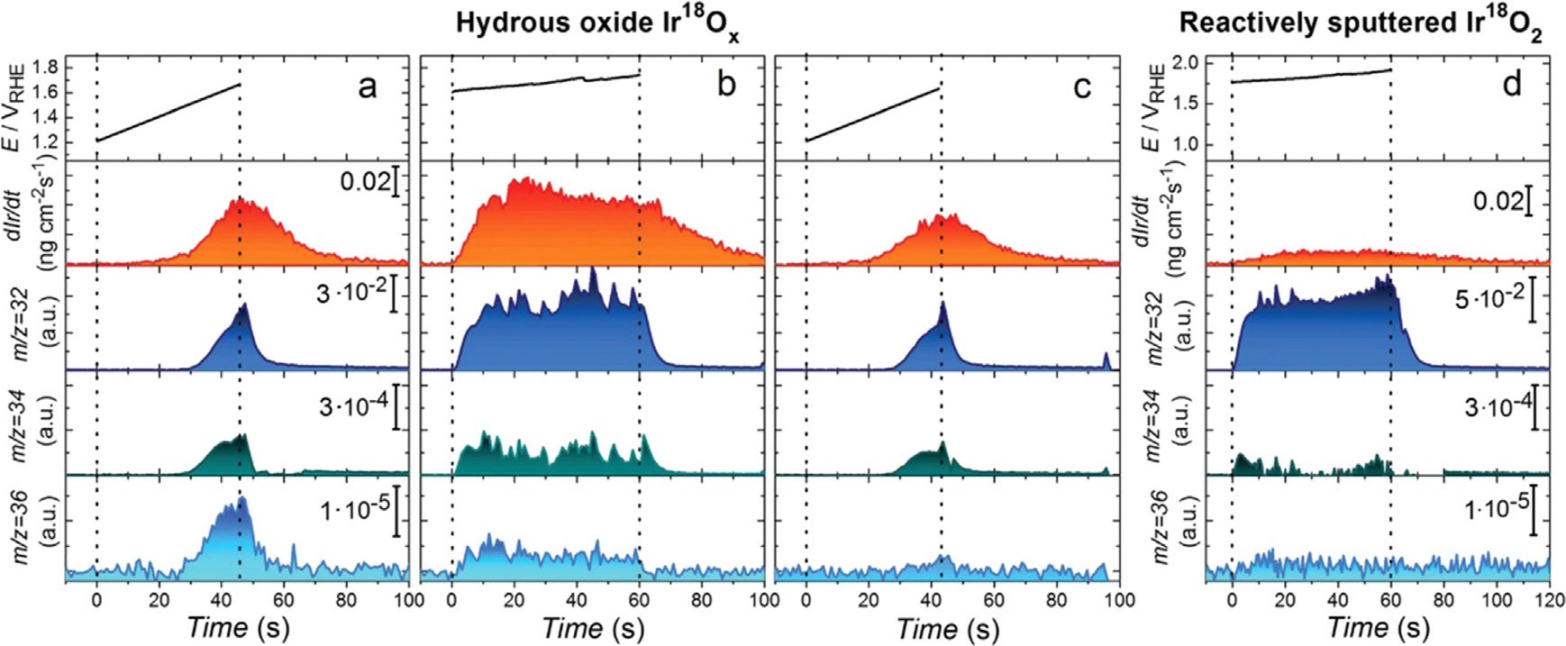
Electrochemical investigation of isotopically labeled hydrous iridium oxide and reactively sputtered iridium oxide through the online dissolution of iridium and the detection of oxygen molecules with *m*/*z* 32 (O^16^O^16^), 34 (O^16^O^18^), and 36 (O^18^O^18^).^[68]^

A study by Kasian et al. further aimed to quantitatively assess the contribution of the oxygen evolved from the lattice to the overall OER.^[68]^ The authors combined SFC‐ICP‐MS and OLEMS measurements with the atomic‐scale structural characterization technique atom probe tomography (APT), which was previously used to unveil the structure of electrochemically grown iridium oxide under galvanostatic conditions.^[70]^ Hydrous Ir^18^O_*x*_ and reactively sputtered Ir^18^O_2_ films were used as model systems in this study. Figure 6 shows the results of the electrochemical experiment together with in situ dissolution measurements and the evolution of volatile products with *m*/*z* 32, 34, and 36. Oxygen with *m*/*z* 32 evolves through a classical adsorbate route and does not involve lattice oxygen. Products with higher *m*/*z* values, in contrast, contain one (*m*/*z* 34, O^16^O^18^) or two (*m*/*z* 36, O^18^O^18^) oxygen atoms from the lattice. Detection of these last two molecules in measurements on hydrous oxide directly confirm the participation of lattice oxygen atoms, which results in the destabilization of the oxide structure and its higher dissolution, compared to reactively sputtered iridium oxide. In the latter case, the dissolution was an order of magnitude lower with a negligible concentration of oxygen evolved through the participation of lattice oxygen atoms. This conclusion could, however, originate from technical limitations arising from the concentrations of the evolved species being below the detection limit. APT analysis of both oxides was used to correlate the difference in the structure and stability and revealed that hydrous oxide nanopores are covered with a layer consisting of Ir‐O and OH in an approximate 1:1 ratio. This finding suggests that hydrous iridium oxide consists of Ir^III^‐OOH species, which can by themselves act as the OER precursor. Indeed, it was found that the ratio between the dissolution and the evolution of oxygen through a recombination of two lattice oxygen atoms was constant, which directly confirms their previously suggested correlation. Additionally, OH groups present in the hydrous layer stabilize the Ir^III^ species in the oxide which can serve as the precursor for the peroxide route, thereby leading to evolution of O^16^O^18^. On the basis of the APT experiments, it can be suggested that the Ir^III^‐OOH species could actually be the degradation intermediate HIrO_2_ (Figure 4) and that its degradation may be accompanied by the release of an oxygen molecule. In reactively sputtered oxide, however, the presence of such species was not detected, which is in line with its higher stability. Quantitatively, on hydrous iridium oxide, approximately 0.05 % of all oxygen molecules are produced by the peroxide route, whereas only 0.01 % of the oxygen molecules originate from the recombination of two oxygen atoms from the lattice.

### 4.2 Lattice Oxygen Evolution Reaction and Stability of Rutile IrO_2_

As mentioned previously, the technical limitations of techniques such as OLEMS can lead to inaccurate conclusions when investigating more‐stable oxides. The studies highlighted in the previous sections have in general concluded that the rutile lattice does not participate in the OER, which could explain its higher stability.[^66^, ^68^] However, to overcome possible detection‐related limitations and test whether the exchange of oxygen anions is nevertheless possible in the case of more rigid structures, a different approach was used in the study reported by Schweinar et al.^[71]^ Instead of detecting the evolved oxygen, the authors used an isotope‐labeled reactively sputtered iridium oxide thin film, anodically polarized it at 1 mA cm^−2^ for 10 minutes in a non‐labeled electrolyte, and afterwards estimated the proportion of exchanged oxygen atoms by APT. The analysis revealed a significant increase in the O^16^ species in the top 2.5 nm of the film, which was direct confirmation of the active involvement of the lattice in the reaction. The overall electrochemically active volume of the catalyst was nonetheless significantly lower than in hydrous iridium oxide, which explains the higher stability of rutile. The active involvement of oxygen from the rutile lattice was recently corroborated in a dynamic OER operation through observation of an increased Ir‐Ir interaction in IrO_2_ by in‐operando XAS measurements.^[72]^ After the release of oxygen from the lattice, the rearrangement of the structure leads to a decreased distance between the Ir atoms, which was proposed to be the origin of the higher stability of rutile. Structural changes in the lattice also lead to stronger Ir−O bonds, which could explain the lower activity of IrO_2_. This is in line with the DFT calculations by Man et al.^[73]^ which showed that the origin of the overpotential in Ir is the very strong binding of O. For a more thorough understanding of the effect of surface orientation on stability and its effect on the participation of the oxide in the reaction, studies on the single‐crystalline models, mostly carried out to date on ruthenium oxide surfaces,^[74]^ should in the future be carried out on iridium oxides.[^75^, ^76^]

## 5 Concluding Remarks and Perspective

**Figure 7:**
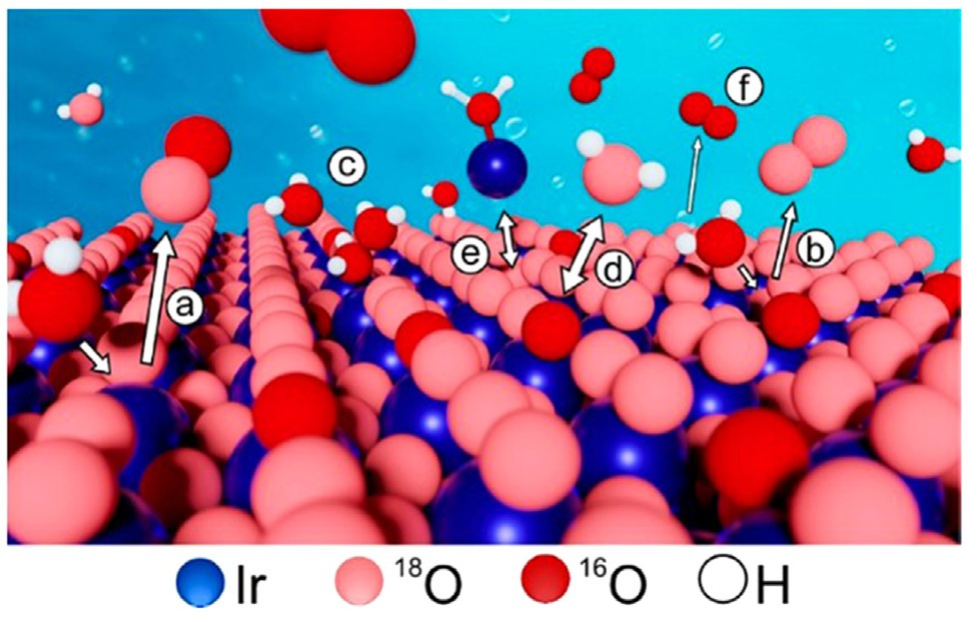
Processes on the surface of iridium oxide catalysts under OER conditions.^[71]^ Copyright 2020, American Chemical Society.

After almost a decade of fundamental dissolution‐oriented studies on model systems, such as metallic iridium disk or thin films, the processes driving the degradation of OER catalysts are now generally well‐understood. Regardless of its nature, the conditions of the OER generally affect the stability of the oxide structure, although not to the same extent. Figure 7 summarizes different processes that trigger the dissolution.^[71]^ The participation of either one (a) or two (b) oxygen atoms in the OER result in the destabilization of the lattice. This occurs more frequently on amorphous oxides, as their structure is more flexible, with a considerably larger catalytically active volume with intercalated water molecules and the occupancy of activated oxygen atoms and Ir^III^‐OOH species, which can by themselves act as OER precursors. In reactively sputtered oxides, the kinetics of lattice oxygen exchange is slower, but nevertheless present. It was previously suggested that the rate of oxygen exchange could potentially serve as a metric for evaluating the stability of different oxides.^[71]^ The vacancies that are created after the removal of oxygen can be refilled through either the adsorption of a water molecule or the migration of bulk oxygen atoms. This inevitably results in surface reconstruction that can further enhance dissolution, for example, through the oxidation of defects (c). Oxygen from the lattice can also be exchanged by oxygen from the water (d). This process is not expected to be particularly destructive; however, it still requires bond rupture and formation, which would lead to destabilization of the surface. The formation of the unstable Ir^III^ species under the OER conditions results in dissolution of this intermediate. Afterwards, the dissolved species can be redeposited back to the surface (e) and thus boost the amorphization of the surface and increase the possibility for further dissolution. Despite various surface processes taking place during the OER, dissolution measurements have, however, shown that the OER occurs on iridium‐based catalysts, regardless of the structure, predominantly through the decomposition of water (f); this makes iridium‐based catalysts currently the catalysts of choice for the OER in acidic media.

**Figure 8:**
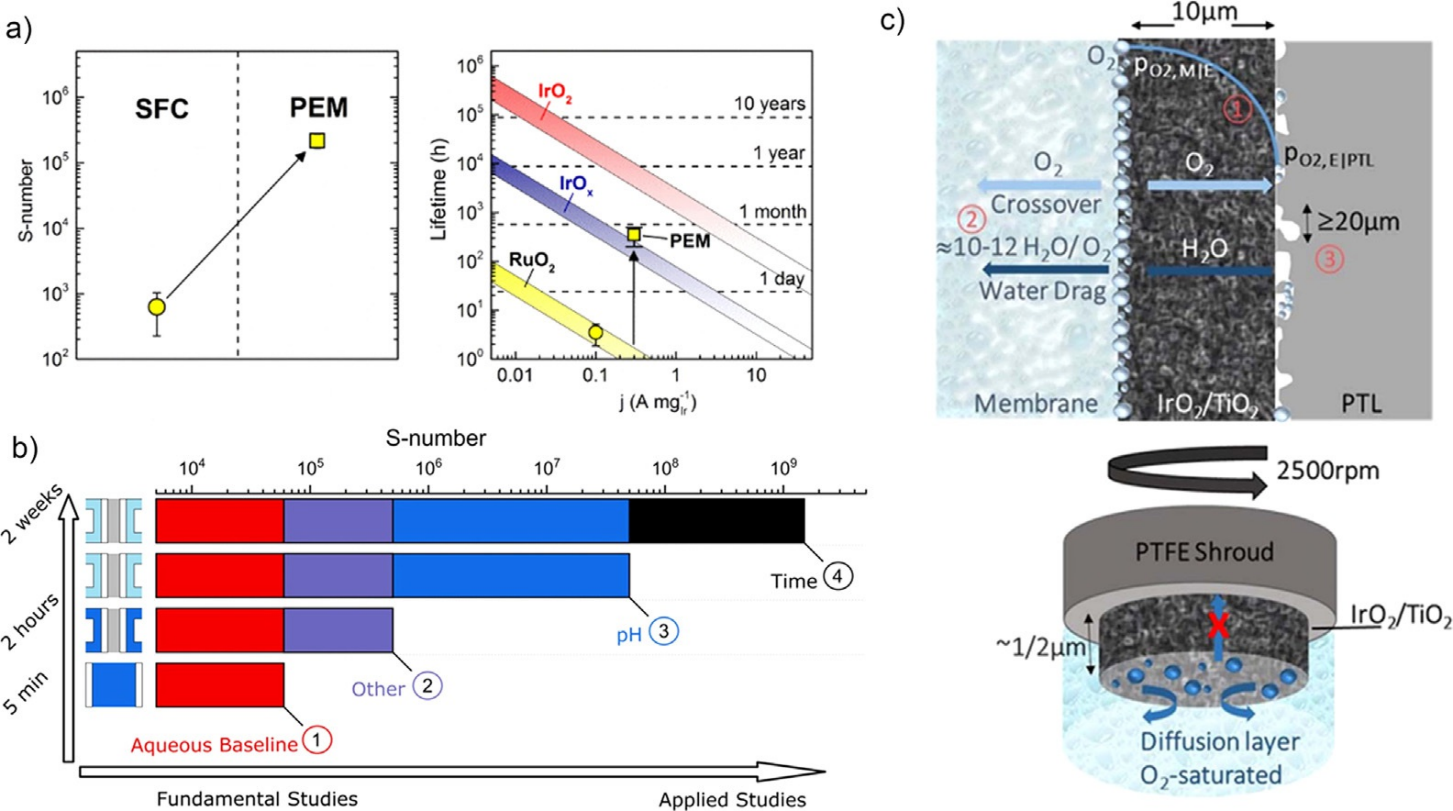
a) Comparison between S‐numbers obtained in aqueous electrolytes by SCF‐ICP‐MS measurements and extracted from the PEM stack.^[47]^ b) Effect of pH and stabilization over time on the calculated S‐number in an aqueous model system and MEA.^[79]^ c) Difference in bubble accumulation in RDE and MEA during OER.^[98]^

However, the question still remains: can the acquired knowledge now be transferred to non‐model systems?[^77^, ^78^] When comparing the S‐numbers of anhydrous ruthenium oxide, measured in either aqueous electrolyte by SFC‐ICP‐MS or extracted from the PEM stack, the calculated lifetimes differed by more than two orders of magnitude. This suggests that dissolution measurements generally overestimate the dissolution of catalysts in the OER (Figure 8 a).^[47]^ This could originate from a different acidity in the PEM stack compared to the half‐cell investigations, where an electrolyte with pH 1 is generally used. Additionally, the diffusivity of dissolved ions out of the membrane or their deposition in the membrane or cathode could result in a lower dissolution of the catalyst. First attempts to evaluate the effect of different parameters on the dissolution rate were presented in a study recently published by Knöppel et al.^[79]^ The study aimed to test different parameters that differ between the model aqueous system and membrane electrode assembly (MEA). The results revealed that the main source of the higher dissolution in model systems is the overestimated acidity and the stabilization in real devices over time (Figure 8 b).

These results confirm the effect of the pH value on the dissolution of iridium under OER conditions. As was discussed above, the instability of Ir^III^ intermediates in the OER and dissolution pathways on more active catalysts leads to an enhanced dissolution of these materials compared to rutile IrO_2_. The observed suppressed dissolution at higher pH values implies that the stability of this intermediate could be pH‐dependent. Future studies should, therefore, focus on the effect of acidity on the stability and activity of iridium‐based catalysts. Although the effect of acidity on the activity was previously shown for various OER catalysts,[^80^, ^81^, ^82^] the stability was overlooked. Furthermore, a deeper understanding of the effective pH value under working conditions in the PEM electrolyzer should be obtained.

Results showcasing the discrepancies between model and real systems pave the way for future studies on the topic, which should aim to close the gap between them. Although we believe that half‐cell measurements in aqueous systems can be used to estimate the stability of not only Ir‐based novel materials, techniques resembling a more realistic environment of MEAs should be concomitantly developed for the evaluation of parameters, such as loading effect, binder content, and impact of 3D architecture, that affect the performance of the catalyst. Moreover, testing under realistic current densities is also desired, but currently unavailable with traditional techniques such as RDE. Setups such as the gas diffusion electrode (GDE) could be used for such studies,[^83^, ^84^] as recently employed for the evaluation of OER catalysts.^[84]^ However, realistic current densities have not yet been achieved, predominantly because of the mass transport limitations that still need to be overcome.

It was shown that stability predominantly depends on the structure of the catalysts. This is especially crucial when nanoparticles with more surface defects such as vacancies, steps, kinks, and grain boundaries are considered. Their effect on the dissolution should, therefore, be more thoroughly studied in future studies.^[85]^ A dissolution study by Jovanovič et al.^[86]^ on different iridium‐based nanoparticles showed some discrepancy from the disk measurements carried out by Cherevko et al.,[^11^, ^55^] which was attributed to a possible particle size effect. The long‐term stability of even crystalline IrO_2_ nanoparticles could potentially be questioned, as Schweinar et al.^[71]^ showed that the top 2.5 nm is actively involved in the OER, which could be detrimental for nanoparticles. As the nanoparticles are often anchored on the conductive oxide supports, their dissolution behavior can be additionally altered through strong metal–support interactions (SMSIs).^[87]^ This effect is increasingly gaining attention as it can minimize Ir dissolution.[^53^, ^88^] It is, however, of immense importance to also consider the corrosion resistance and conductivity of the support material, as it has been demonstrated that it can affect both the activity and stability of catalytic material.[^89^, ^90^, ^91^] Advanced electron microscopy techniques, such as in situ transmission electron microscopy (TEM)^[92]^ and identical location TEM (IL‐TEM),^[93]^ should be developed to observe compositional, structural, and morphological changes in the nanoparticles during the OER. Whereas the unambiguous interpretation of the obtained data is limited predominantly by the interaction of the electron beam with the electrolyte in liquid in situ TEM, IL‐TEM is now a generally well‐established technique for the atomic‐scale observation of nanoparticulate electrocatalysts.[^94^, ^95^] Only recently a novel method, namely a modified floating electrode (MFE), was developed and applied to the oxygen reduction reaction (ORR). This technique enables facile handling of the delicate TEM grids and operation under realistic current densities.^[96]^ The challenge which still needs to be overcome to efficiently use MFE, GDE, or TF‐RDE for studying the OER is efficient removal of the generated oxygen bubbles. Their effect was shown in publications by El‐Sayed and co‐workers.[^44^, ^97^, ^98^] In their most recent contribution, the authors attributed the more efficient removal of bubbles in MEA to the generation of an O_2_ pressure gradient in the membrane/electrode/porous transport layer (PTL) interface and electro‐osmotic drag of water to the membrane, which cannot be stimulated in an RDE setup because of different configurations (Figure 8 c). Thus, to design an aqueous system where realistic current densities could be reached, these dynamic processes in the catalyst layer should be stimulated.

## Conflict of interest

The authors declare no conflict of interest.

## Biographical Information

*Anja Lončar obtained her master's degree (2020) in Chemistry at the University of Ljubljana, Slovenia. During this time, she was a visiting student in the Electrochemical Energy Conversion group of Helmholtz Institute Erlangen‐Nürnberg for Renewable Energy (HI‐ERN), Germany, led by Dr. Serhiy Cherevko. She is currently a PhD candidate with Prof. Nejc Hodnik at the Laboratory of Electrocatalysis at the National Institute of Chemistry in Ljubljana (Slovenia). Her studies focus on investigating the stability of nanoparticulate iridium‐based electrocatalysts for the oxygen evolution reaction by advanced electrochemical methods*.

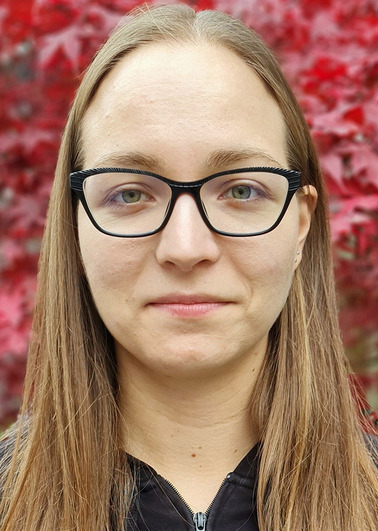

## Biographical Information

*Daniel Escalera‐López completed his PhD with integrated studies in 2019 on “Fuel cells and their Fuels” at the Chemical Engineering department from the University of Birmingham (United Kingdom), with Dr. Neil V. Rees. Since 2019, he has been a postdoctoral researcher in the Electrochemical Energy Conversion team of the Helmholtz Institute Erlangen‐Nürnberg for Renewable Energy (HI‐ERN), Germany. His research involves the study of activity‐stability relationships of noble and non‐noble metal‐based catalysts for electrochemical water splitting by in situ and in operando coupled electrochemical and surface characterization techniques*.

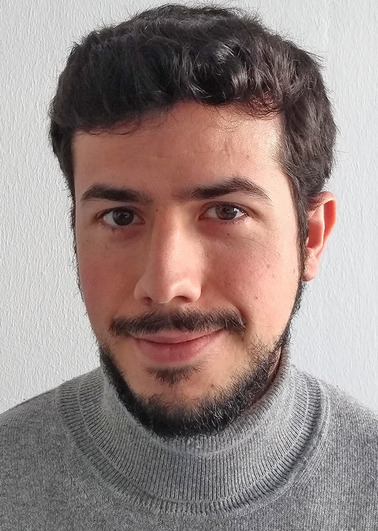

## Biographical Information

*Serhiy Cherevko received his PhD with Prof. Chan‐Hwa Chung in Nanoscience at the Sungkyunkwan University, South Korea, in 2009. He then carried out postdoctoral research in chemical engineering at Sungkyunkwan University (2009–2011) and in the department of Interface Chemistry and Surface Engineering, Max‐Planck Institut für Eisenforschung, Germany (2011–2015). Since 2016, he has been head of the Electrochemical Energy Conversion group of the Helmholtz Institute Erlangen‐Nürnberg for Renewable Energy (HI‐ERN), Germany. His research focuses on fuel cells, water electrolysis, photo‐electrochemistry, and recycling noble metals*.

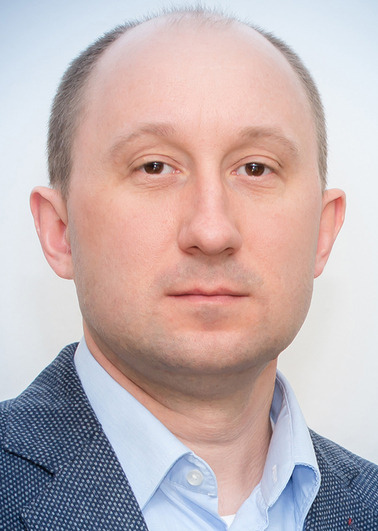

## Biographical Information

*Nejc Hodnik completed his PhD with Dr. Stanko Hočevar in 2013 at the National Institute of Chemistry (NIC). He was then a Marie Curie Intra‐European fellow associated with the group of Prof. Mayrhofer in the MPIE. In 2019, he became associate professor at the University of Nova Gorica, obtained an ERC Starting Grant project, and started a new Laboratory of Electrocatalysis. His research focuses on nanoscale electrocatalyst degradation studies with advanced electrochemical characterization methods, synthesis, electron microscopy, recycling, etc*.